# Atlantoaxial Dislocation Presenting with Dizziness

**DOI:** 10.3390/diagnostics16131949

**Published:** 2026-06-23

**Authors:** Seoyeon Kim, Ji-Soo Kim, Jin Sup Yeom, Ngoc Quyen Nguyen, Beomseok Jeon, Hyo-Jung Kim

**Affiliations:** 1 Department of Neurology, Inha University Hospital, Inha University College of Medicine, Incheon 22332, Republic of Korea; pivoinerosee@gmail.com; 2 Department of Neurology, Seoul National University College of Medicine, Seoul 03080, Republic of Korea; jisookim@snu.ac.kr; 3 Dizziness Center, Clinical Neuroscience Center, Department of Neurology, Seoul National University Bundang Hospital, Seongnam 13620, Republic of Korea; 4 Department of Orthopaedic Surgery, Seoul National University College of Medicine, Seoul National University Bundang Hospital, Seoul 13620, Republic of Korea; 5 Spine Surgery Unit, Polyclinic and Premier Healthcare Center, 108 Military Central Hospital, Hanoi 100000, Vietnam; 6 Department of Neurology, BJ Center for Comprehensive Parkinson Care and Rare Movement Disorders, Chung-Ang University Health Care System Hyundae Hospital, Namyangju 12013, Republic of Korea; 7 Biomedical Research Institute, Seoul National University Bundang Hospital, Seongnam 13620, Republic of Korea

**Keywords:** atlantoaxial dislocation, atlantoaxial subluxation, cervical dizziness, vertigo, torticollis, cervical spine, bow hunter’s syndrome

## Abstract

Atlantoaxial dislocation (AAD) or subluxation (AAS) is a potentially life-threatening disorder caused by instability between the atlas and axis. Typical symptoms include neck pain, torticollis, and neurological deficits, but dizziness has rarely been reported in association with AAD/AAS. We describe an adolescent girl who presented with non-spinning dizziness, neck pain, and torticollis after prolonged head flexion and extension. Neurological examination showed rightward torticollis without nystagmus. Cervical spine imaging revealed atlantoaxial rotatory dislocation and fixation, while CT angiography confirmed patency of both vertebral arteries. Conservative management with cervical traction was ineffective, and surgical reduction with C1–C2 fixation and fusion was performed. The patient experienced complete resolution of dizziness and torticollis postoperatively without complications. This image-based report describes a rare case of AAD in which dizziness was the main presenting symptom. This case highlights that cervical instability should be considered in the differential diagnosis of dizziness.

**Figure 1:**
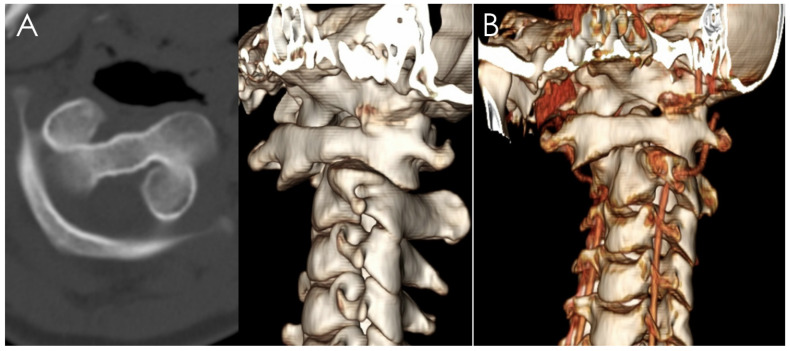
**Atlantoaxial rotatory dislocation presenting with dizziness in an adolescent girl.** (**A**) Axial and three-dimensional reconstructed cervical spine CT images show C1–C2 rotatory fixation with bilaterally locked facets; the lateral mass of the atlas is rotated relative to the odontoid process, and the normal anatomical alignment between C1 and C2 is lost. (**B**) Cervical CT angiography demonstrates patent vertebral arteries in the presence of atlantoaxial dislocation. A previously healthy adolescent girl presented with dizziness, neck pain, and torticollis for two months. Just after a prolonged head-flexed posture for a nap during a long bus trip, and a subsequent head-extended posture while seated, she began to suffer from non-spinning dizziness and neck pain that worsened with neck extension. She denied any family history of dystonia or other movement disorders. General physical and neurological examinations were unremarkable except for rightward torticollis. On examination, there was no spontaneous, gaze-evoked, vibration-induced, or head-shaking nystagmus. Head rotation and extension while seated provoked no nystagmus. Video-oculography documented normal saccades and smooth pursuit. Ocular alignment was normal without skew deviation. Romberg test and tandem gait were intact. Caloric testing and vestibular-evoked myogenic potentials were not performed, and the video head impulse test could not be obtained because of neck pain. Review of the cervical MRIs obtained at an outside hospital had disclosed a suspicious malalignment between the atlas and axis along with torticollis. Additional cervical spine radiographs and CT scan showed an atlantoaxial rotatory dislocation and fixation (Figure 1A). Cervical spine CT angiography documented patent vertebral arteries on both sides (Figure 1B). Because conservative management with 16-pound Gardner–Wells tong traction for seven days was ineffective, she underwent open reduction in the rotatory dislocation with C1–C2 fixation using bilateral C1 posterior-arch and C2 pedicle screws and fusion. The operation resulted in complete resolution of both dizziness and torticollis without any complications, and the patient remained symptom-free until the last follow-up, 42 months after the surgery. Although there have been considerable debates on the concept of ‘cervical or cervicogenic dizziness’, dizziness is occasionally reported in association with cervical pain, whiplash injury, degenerative cervical spondylosis and other cervical disorders [1]. The cervical afferents provide inputs to the secondary vestibular neurons in the vestibular nuclear complex, and proprioceptive signals from the neck are an integral component of the vestibular system [2]. Thus, abnormal sensory information from deranged joint receptors or muscle proprioceptors in the upper cervical regions, among many others, has been proposed as a mechanism of cervical dizziness [3,4]. However, the pathogenesis of cervical dizziness still requires further elucidation with high quality data from well-designed studies [5]. Even with these controversies, the consistent features of cervical dizziness include (1) neck stiffness and pain aggravated during neck movements, (2) transient imbalance, light-headedness, or illusory self-motion triggered by neck movements, and (3) improvement of neck pain, neck stiffness and dizziness by neck-directed therapy [5]. All these features were also observed in our patient. Vestibular migraine, a common cause of dizziness in adolescent girls, was considered unlikely given the absence of any history of headache and the mechanically reproducible nature of the symptoms. Given that the dizziness in our patient was aggravated with head extension, compression of the vertebral artery may be considered a mechanism of dizziness during neck motion [6], also known as bow hunter’s syndrome, a rare type of symptomatic vertebrobasilar ischemia resulting from compression of the vertebral artery during head motion [7]. Patients with this condition may show nystagmus along with vertigo during the attacks triggered by head rotation [8]. Because of its anatomic susceptibility and winding pathway, mechanical compression of the vertebral artery may occur when the head is rotated [9]. Indeed, patients with AAD may develop posterior circulation stroke as a complication [10]. However, most patients with vertebral artery compression syndrome show a stenosis or anomaly of the vertebral artery on one side and compression of the dominant vertebral artery during contralateral head rotation even though compression of both vertebral arteries has rarely been documented [11]. In contrast, our patient showed the patency of both vertebral arteries on CT angiography without any nystagmus during head rotation or extension while sitting. Since the patient did not have dynamic angiography to exclude compression of the vertebral arteries during head rotation, vertebral artery compression syndrome was only partially excluded, which remains a limitation of this report. Importantly, this case underscores that dizziness accompanied by neck pain and torticollis should prompt careful evaluation of the upper cervical spine.

## Data Availability

Anonymized data supporting the findings of this case will be shared by the corresponding author upon reasonable request from any qualified investigator.
